# Inhibition Effect of Triphenylmethane Dyes for the Corrosion of Carbon Steel in CO_2_-Saturated NaCl Corrosion Medium

**DOI:** 10.3390/ma17051094

**Published:** 2024-02-28

**Authors:** Lincai Peng, Shaomu Wen, Jing Yan, Huali Yu, Zhan Wen, Zhi Wang

**Affiliations:** 1 Research Institute of Natural Gas Technology, PetroChina Southwest Oil and Gasfield Company, Chengdu 610051, China; 2 National Energy R&D Center of High Sulfur Gas Exploitation, Chengdu 610213, China; 3 High Sulfur Gas Exploitation Pilot Test Center, Chengdu 610213, China; 4 PetroChina Southwest Oil and Gasfield Company, Chengdu 610051, Chinawangzhi@petrochina.com.cn (Z.W.); 5 Sichuan Changning Natural Gas Development Co., Ltd., Chengdu 610065, China

**Keywords:** CO_2_ corrosion, triphenylmethane dyes, corrosion inhibitor, carbon steel, quantum chemical calculations, molecular dynamics simulation

## Abstract

Carbon dioxide corrosion presents a significant challenge in the oil and gas field. This study simulates the corrosive environment characteristics of oil and gas fields to investigate the corrosion inhibition properties of three triphenylmethane dyes. The inhibitive performance and mechanisms of these dyes were analyzed through weight loss and electrochemical testing, revealing that crystal violet (CV) exhibited a superior inhibition effectiveness over malachite green (MG) and Fuchsine basic (FB). At a concentration of 150 ppm in a CO_2_-saturated 5% NaCl solution at 25 °C, CV achieved an impressive maximum inhibition efficiency of 94.89%. With the increase in temperature, the corrosion rate slightly decreased, and the corrosion rate was 92.94% at 60 °C. The investigated CV acted as a mixed-type corrosion inhibitor and its protection obeyed the Langmuir adsorption isotherm. The corrosion morphology was characterized by scanning electron microscopy (SEM), X-ray photoelectron spectroscopy (XPS), and confocal laser scanning microscopy (CLMS). Quantum chemical calculations and molecular dynamics simulations were employed to validate the corrosion inhibition mechanisms, providing guidance for the further application of these dyes in corrosion control.

## 1. Introduction

Corrosion is a serious problem that significantly impacts industrial development, particularly in the oil and gas industry, where it has caused serious economic losses and potential safety hazards [1,2]. One of the most common types of corrosion is CO_2_ corrosion, which forms an acidic medium that corrodes the metal surface, leading to equipment damage and production interruptions [3]. The production and transportation processes of oil and gas often face severe CO_2_ corrosion issues, impeding the industry’s safe and efficient production. To address the corrosion problem, the injection of corrosion inhibitors is considered the most economically effective approach [4,5,6].

Most dyes contain complex molecular structures with both hydrophilic and hydrophobic segments that are associated with many donor centers that interact with the metallic species, which is essential for the corrosion mitigation effect [7,8]. There are some literature studies that show that many dye series have excellent anticorrosive properties for many metal/electrolyte combinations because of their abundant electron-donating sites as polydentate and chelating ligands [9,10,11]. Triphenylmethane dyes are compounds with abundant phenyl ring structures which generally have strong interactions with the metal surface [12]. They have a good solubility and adsorption capacity, allowing them to quickly attach to the metal surface and form a protective film that hinders the attack of the corrosive medium. The inhibition of type 304LSS corrosion in hydrochloric acid by New Fuchsin was explored in the study of Zahed et al. [13]. New Fuchsin has been found to be a good, mixed-type inhibitor of corrosion, which decreases the partial cathodic and anodic current densities. Li et al. studied the inhibition effect of crystal violet (CV) on the corrosion of cold-rolled steels in 1.0 M HCl solution and found that it is an excellent inhibitor [14]. Careful examination of the data reveals that triphenylmethane dyes can block the active sites of corrosion and reduce the corrosion current density, thereby inhibiting the corrosion of metal in hydrochloric acid [15,16,17]. Despite numerous studies on dyes as corrosion inhibitors, the performance of triphenylmethane dyes in CO_2_ corrosion remains unexplored, which represents a novel direction for corrosion research.

In this study, we innovatively investigated the potential of triarylmethane dyes as corrosion inhibitors in CO_2_-rich environments that simulate the corrosive environment in the oil and gas industry. Quantum chemical calculations and molecular dynamics simulations were employed to elucidate the underlying corrosion mechanisms. By focusing on a class of compounds known for their strong adsorption abilities and electron-donating characteristics, we tried to open up new avenues for the development of corrosion inhibitors with enhanced performance and sustainability.

## 2. Materials and Methods

### 2.1. Materials

Sodium chloride, malachite green (MG), crystal violet (CV), Fuchsine basic (FB) were purchased from Aladdin Chemical Reagent Co., Ltd. (Shanghai, China) with an aluminum mass fraction purity of 98%. Deionized water purified by Milli-Q filtration was used for all the experiments. All corrosion tests were performed on L360N carbon steel with a size of 3 cm × 1.5 cm × 0.3 cm and were obtained from Shandong Yangxin Shengxin Technology Co., Ltd. (Shandong, Jinan, China), and their chemical composition was basically as follows (%, wt): C 0.2, Si 0.35, Mn 1.42, S 0.019, P 0.02, and balanced Fe. All specimens were consistently ground with 600, 800, and 1200-grit SiC papers, rinsed with distilled water, degreased in acetone, washed with anhydrous ethanol, dried, weighed (with an accuracy of 0.0001 g), and stored in a desiccator until use.

### 2.2. Weight Loss Measurements

All specimens were exposed to CO_2_-saturated water with 5% NaCl brine solution containing 100 ppm dyes for three days at 40 °C. Three specimens were tested for each condition simultaneously. After the tests, specimens were taken out and corrosion products on the surface of specimens were removed with a Clark’s solution (10 g hexamethylenetetramine, 100 mL hydrochloric acid, and 900 mL water) [18]. Then, these samples were rinsed in water, cleaned in anhydrous ethanol, dried, and weighed. The corrosion rate (ν, mm/y) and inhibition efficiency (η, %) can be calculated by Equations (1) and (2):

(1) $$\nu =\frac{8.76\times 10^{4}\times (m_{1}-m_{2})}{A\times \rho \times t}$$

where *m*_1_ and *m*_2_ represent the weights of specimens before and after the experiment, respectively (g); *A* represents the surface area (cm^2^); *ρ* represents the density of the specimen (g/cm^3^); and *t* represents the experimental duration (h).

(2) $$\eta \left(\%\right)=\frac{\nu_{0}-\nu_{1}}{\nu_{0}}\times 100$$

where *ν*_0_ and *ν*_1_ represent the corrosion rates of the specimens in the absence and presence of inhibitors, respectively.

### 2.3. Electrochemical Measurements

All electrochemical assays were conducted on a three-electrode cell in a glass container (250 mL) at allocated temperatures via a CS2350 electrochemical workstation (Wuhan Corrtest Instruments Corp., Ltd., Wuhan, China), in which L360N carbon steel with a tested surface area of 0.5 cm^2^, a platinum sheet, and saturated calomel electrode (SCE) behaved as the working, counter-, and reference electrodes, respectively. All the tests were performed in a CO_2_-saturated solution with 5% NaCl containing various concentrations of inhibitors at different temperatures. After, samples were immersed in the solution to reach steady state with the measurement of the open-circuit potential (OCP). Electrochemical impedance spectra (EIS) were obtained in the frequency range from 10^5^ to 10^−2^ Hz with a sinusoidal wave amplitude of 5 mV. Following the measurement, the relevant test data were fitted to obtain equivalent circuit models with ZSimpWin software v3.60. Potentiodynamic polarization (PDP) tests were performed in the potential bounds of −0.25~0.25 V vs. OCP with a sweeping rate of 0.5 mV/s. All of the electrochemical tests were repeated three times to ensure the reproducibility of experimental data.

### 2.4. Surface Analysis

The surface morphology of the specimens was examined by scanning electronic microscopy (SEM, JSM-IT500HR, JEOL, Tokyo, Japan) and confocal laser scanning microscopy (CLSM, VK-150K, Keyence, Osaka, Japan). X-ray photoelectron spectroscopy (XPS, K-Alpha, Thermo Scientific, Waltham, MA, USA) was employed to analyze the surface composition of the samples with a monochromatic Al Kα X-ray source.

### 2.5. Quantum Chemical Calculations

These corrosion inhibitor molecules were optimized with quantum chemical calculations on the basis of density functional theory (DFT) using the B3LYP/6-311++G** method with the consideration of the solvent effects (water) using Gaussian v09W software. The corresponding quantum chemical calculation parameters were obtained to explore the interaction between these dye molecules and steel surface, including the highest occupied molecular orbital (HOMO), the lowest unoccupied molecular orbital (LUMO), and their energy gap (ΔE = ELUMO − EHOMO) [19]. The electrostatic potential (ESP) distribution on a molecular surface was visualized using Visual Molecular Dynamic (VMD) v1.9.3 software [20].

### 2.6. Molecular Dynamics (MD) Simulation

The adsorption behavior of three dye molecules on the iron substrate was calculated by MD simulation with the Forcite module in Materials studio v7.0 software [21]. The Fe (110) surface was chosen to simulate the interaction between the inhibitor and the surface of the specimen due to its highly stabilized and packed structure [22]. The solvent layer (250 H_2_O, 4 Cl^−^, 4 Na^+^, and the optimized structure of inhibitor) was in interaction with the Fe (110) surface in the COMPASS II force field. A vacuum layer of 15 Å was established above the solution layer to isolate the interaction of periodic structures in the vertical direction [23]. MD simulations were conducted under a canonical ensemble (NVT) for 1.0 fs time steps with 500 ps as the simulation time at 298.15 K. In addition, adsorption energy (*E*_ads_) was used to quantitatively evaluate the interaction strength of the inhibitor with the Fe surface and was calculated by Equation (3) [24]:

*E*_ads_ = *E*_total_ − (*E*_surf+solu_ + *E*_inh+solu_) + *E*_solu_ (3)

where *E*_total_ represents the energy of the entire adsorption system, *E*_surf+solu_ represents the single-point energy of the Fe (110) surface and solvent, *E*_inh+solu_ stands for the energy of the inhibitor and solvent, and *E*_solu_ is the energy of the solvent.

## 3. Results and Discussion

### 3.1. Weight Loss Measurements

Weight loss measurement is simple and straightforward to use to evaluate the corrosion of metals [25]. Table 1 lists the results of specimens exposed to the solution with 100 ppm inhibitors for three days at 40 °C. The corrosion rate and inhibition efficiency of the specimens in the solution with CV, MG, and FB are 0.050 mm/y, 0.102 mm/y, and 0.143 mm/y and 82.8%, 64.1%, and 49.8%, respectively, while the average corrosion rate is 0.323 mm/y in the blank solution. The addition of dyes results in an increase in corrosion inhibition efficiency, which suggests that dyes can likely adsorb on the specimen surface. Among them, the corrosion inhibition efficiency of CV is best.

### 3.2. Electrochemical Measurements

Electrochemical measurements are effective methods for exploring corrosion mechanisms at the metal solution interface [26]. In order to further investigate the inhibition properties of CV, EIS and PDP experiments were performed in the solution without or with different concentrations of CV at 25, 40, and 60 °C.

#### 3.2.1. EIS Measurements

**Figure 1:**
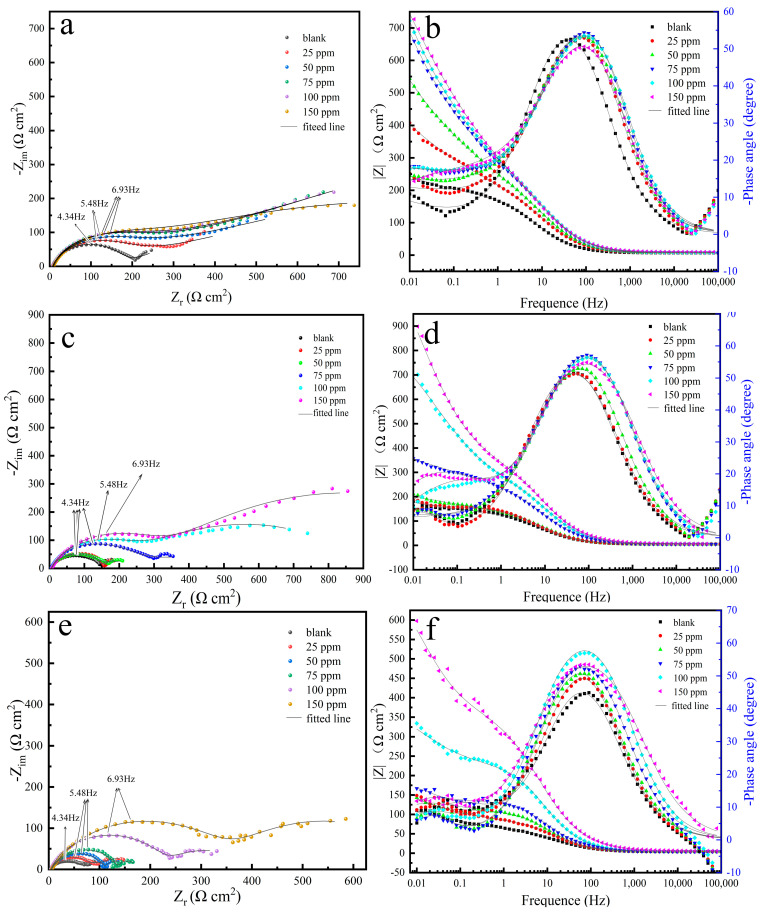
Nyquist (**a**,**c**,**e**) and Bode plots (**b**,**d**,**f**) in the CO_2_−saturated 5% NaCl containing various concentrations of CV at different temperature: (**a**,**b**) 25 °C, (**c**,**d**) 40 °C, (**e**,**f**) 60 °C.

**Figure 2:**
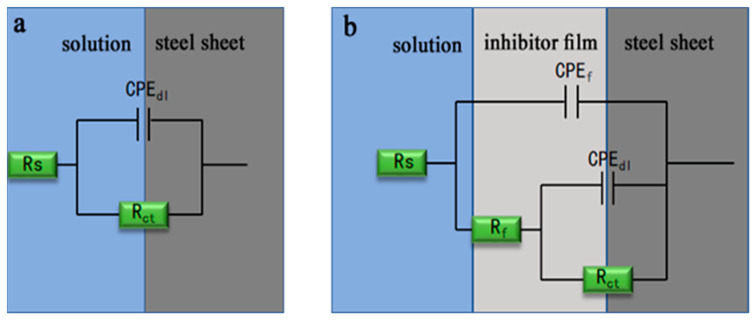
The equivalent circuit models used for fitting the EIS data: (**a**) in the absence of inhibitor, (**b**) in the presence of inhibitor.

The results of the Nyquist and Bode plots of the specimens are shown in Figure 1. The Nyquist plot demonstrates a semicircular arc in the high-frequency domain, which is indicative of the electrode surface’s roughness and non-uniformity as well as the adsorption of corrosion inhibitors on the carbon steel surface. In the low-frequency domain, the presence of a sloped line suggests that a diffusion process of ions is taking place at the electrode [27]. The characteristic frequency ω_c_ of the characteristic arc is labeled with a black arrow in the figure. It can be seen that the shapes of the impedance with various inhibitor concentrations at different temperatures are similar, with all showing a single capacitive arc. The diameter of the semicircle increases considerably with the increase in corrosion inhibitor concentration. However, the diameter or radius gradually decreases under the same corrosion inhibitor concentration as the temperature gradually increases. A higher diameter of the capacitive arc reflects the formation of potential barriers that must be resolved to facilitate charge transfer, suggesting a decrease in the rate of corrosion [28]. Moreover, the wide phase angle peaks in the presence of CV indicate the existence of two time constants compared to the blank solution in the Bode plots, indicating that the corrosion inhibitor has successfully adsorbed on the surface of the steel sheet to form a corresponding adsorption film [25]. The equivalent circuit moles are used to fit the EIS data without or with CV and are shown in Figure 2. It is commonly accepted that the corrosion mechanism of unprotected metals often potentially involves a charge transfer mechanism. However, the introduction of corrosion inhibitors tends to complicate the metal’s corrosion mechanism. Consequently, for the uninhibited solution, an equivalent circuit like that shown in Figure 2a is typically employed for fitting purposes, while for the more intricate impedance characteristics observed after the addition of corrosion inhibitors, equivalent circuits of Figure 2b are used for fitting. In these equivalent circuits, R_s_ represents the solution resistance, R_ct_ represents the charge transfer resistance, CPE_dl_ represents the constant-phase element standing for the double-layer capacitance, and R_f_ and CPE_f_ represent the resistance and capacitance of the corrosion product film and/or the inhibitor films, respectively [29]. The equivalent circuit indicates that the addition of CV results in the formation of a film on the carbon steel surface, thereby inhibiting its corrosion.

#### 3.2.2. PDP Measurements

**Figure 3:**
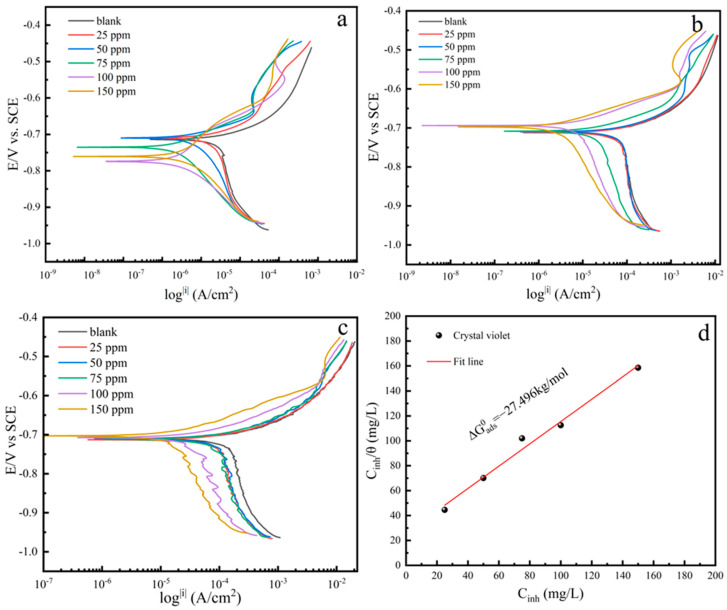
Potentiodynamic polarization curves in the CO_2_−saturated 5% NaCl containing various concentrations of CV at different temperatures: (**a**) 25 °C, (**b**) 40 °C, (**c**) 60 °C, and (**d**) the Langmuir adsorption isotherm at 40 °C.

Figure 3a–c show the polarization curves obtained in blank solution as well as with the addition of different corrosion inhibitor concentrations at temperatures of 25 °C, 40 °C, and 60 °C, respectively. It can be seen from the figures that the addition of the corrosion inhibitor results in a shift in both the cathodic and anodic polarization curves to the left to various extents, and the reduction in polarization current density indicates that the corrosion inhibitor has a significant inhibitory effect on both the anodic dissolution and the reduction in cathodic hydrogen ions in the corrosion process of carbon steel [18]. Additionally, the figures reveal that the corrosion potential of this inhibitor does not show significant differences at different concentrations, indicating that CV is a mixed-type corrosion inhibitor [30].

By fitting the polarization curves, the following electrochemical parameters are obtained: *E*_corr_ (corrosion potential) and *i*_corr_ (corrosion current density), which are listed in Table 2. The inhibition efficiency (*η*_p_ %) of the corrosion inhibitor can be calculated using Equation (4) [29]:

(4) $$\eta_{p}\left(\%\right)=\frac{i_{\mathrm{corr}}^{0}-i_{\mathrm{corr}}}{i_{\mathrm{corr}}^{0}}\times 100$$

where $i_{\mathrm{corr}}^{0}$ and $i_{\mathrm{corr}}$ represent the corrosion current density of the blank solution and the solution with the added corrosion inhibitor, respectively.

**Figure 4:**
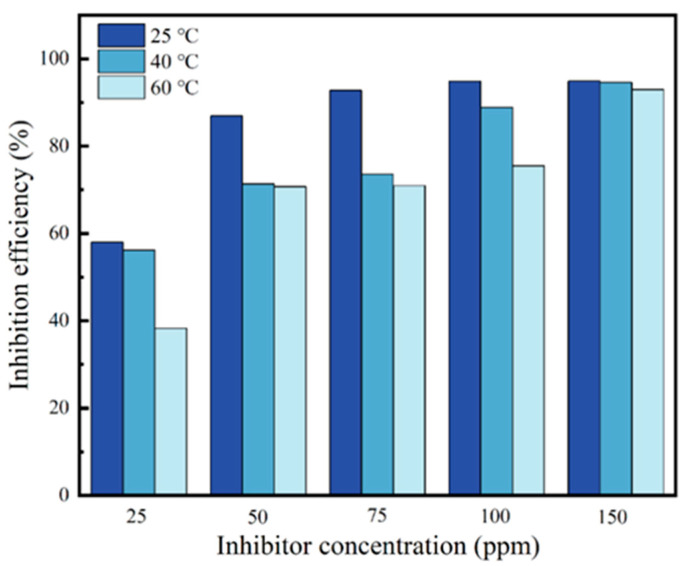
Inhibition efficiencies of L360N carbon steel in the CO_2_−saturated 5% NaCl corrosion medium with different concentrations of CV at 40 °C.

From Table 2, it is evident that *i*_corr_ decreases and *η*_p_ increases with the increase in the concentration of the corrosion inhibitor at different temperatures. Furthermore, the lowest *i*_corr_ and highest *η*_p_ can be observed at 25 °C by comparing these values at different temperatures, indicating that CV is most effective at inhibiting corrosion at this temperature. As the temperature increases, the inhibitory effect of the corrosion inhibitor gradually weakens (as shown in Figure 4). Both the cathodic and anodic current densities are observed to decrease after the addition of the corrosion inhibitor, which indicates that the adsorption of the inhibitor film on the surface hinders both the cathodic and anodic processes [31]. Table 3 lists the inhibition efficiencies of the studied dyes and other dyes reported in the literature. The literature primarily investigates the corrosion inhibition performance in hydrochloric or sulfuric acid solutions, with no studies addressing its efficacy against CO_2_ corrosion. Table 3 reveals that CV exhibits an excellent corrosion inhibition efficiency in the CO_2_-saturated 5% NaCl solution.

### 3.3. Adsorption Thermodynamic and Kinetic Analysis

In order to investigate the adsorption mechanism, the adsorption isotherm of CV is shown in Figure 3d, and its adsorption capacity is calculated for the corrosion inhibitor at 40 °C using the following Langmuir adsorption formula [36]:

(5) $$\frac{C_{\mathrm{inh}}}{\theta }=\frac{1}{K_{\mathrm{ads}}}+C_{\mathrm{inh}}$$

where *C*_inh_ represents the concentration of CV, *θ* is the coverage rate of the corrosion inhibitor on the steel surface (*θ* = η_p_), and *K*_ads_ is the adsorption–desorption equilibrium constant, which could be determined with the intercept of the *C*_inh_/*θ* vs. *C*_inh_ plots.

The standard Gibbs free energy of adsorption ($∆G_{\mathrm{abs}}^{0}$) can be calculated by Formula (6) [37]:

(6) $$∆G_{\mathrm{ads}}^{0}=-\mathrm{RTln}(1\times 10^{6}K_{\mathrm{ads}})$$

where R represents the universal gas constant (8.314 JK^−1^mol^−1^), T is the thermodynamic temperature, and $1\times 10^{6}$ is the value of the concentration of water (mg/L) in solution.

The thermodynamic parameters of CV are listed in Table 4. The linear correlation coefficient (R^2^) is 0.99, demonstrating that CV follows the Langmuir adsorption model. As calculated, the Gibbs free energy ($∆G_{\mathrm{abs}}^{0}$) of the corrosion inhibitor is −27.496 kJ/mol, indicating that the adsorption type of this adsorbent simultaneously exhibits physical adsorption and chemical adsorption [25].

**Figure 5:**
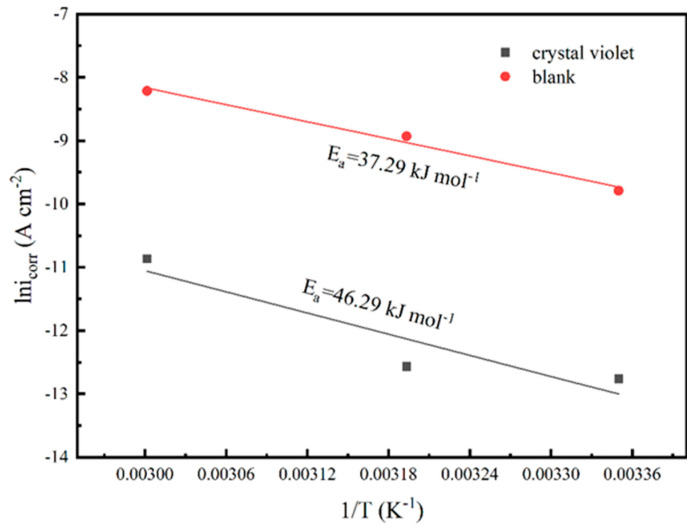
Arrhenius plots of L360N carbon steel in the CO_2_−saturated 5% NaCl corrosion medium with and without 150 ppm inhibitor of MV at 40 °C.

Taking into consideration the impact of temperature on the corrosion inhibitor, the corrosion kinetics were further investigated in this process. The relationship between current density and temperature was explored in a solution containing the inhibitor or an inhibitor-free solution using the Arrhenius equation [38]:

(7) $$i_{corr}=Aexp(-\frac{E_{a}}{RT})$$

where *A* represents the frequency factor, E_a_ is the activation energy, and i_corr_ is the current density of the corrosion inhibitor at 150 ppm. R and *T* represent the universal gas constant and thermodynamic temperature, respectively. The Arrhenius plots and E_a_ are shown in Figure 5. As can be seen from Figure 5, the activation energy with the addition of CV (*E*_a_ = 46.29) is higher than that of without the inhibitor (*E*_a_ = 37.29). This result suggests that the addition of the corrosion inhibitor increases the energy barrier of the corrosion reaction, inhibiting the occurrence of the reaction. Generally speaking, the higher the barrier, the less likely the reaction is to proceed [18]. Therefore, it can be seen that the addition of CV effectively inhibits the corrosion of carbon steel.

### 3.4. Surface Characterization

#### 3.4.1. Surface Morphology

**Figure 6:**
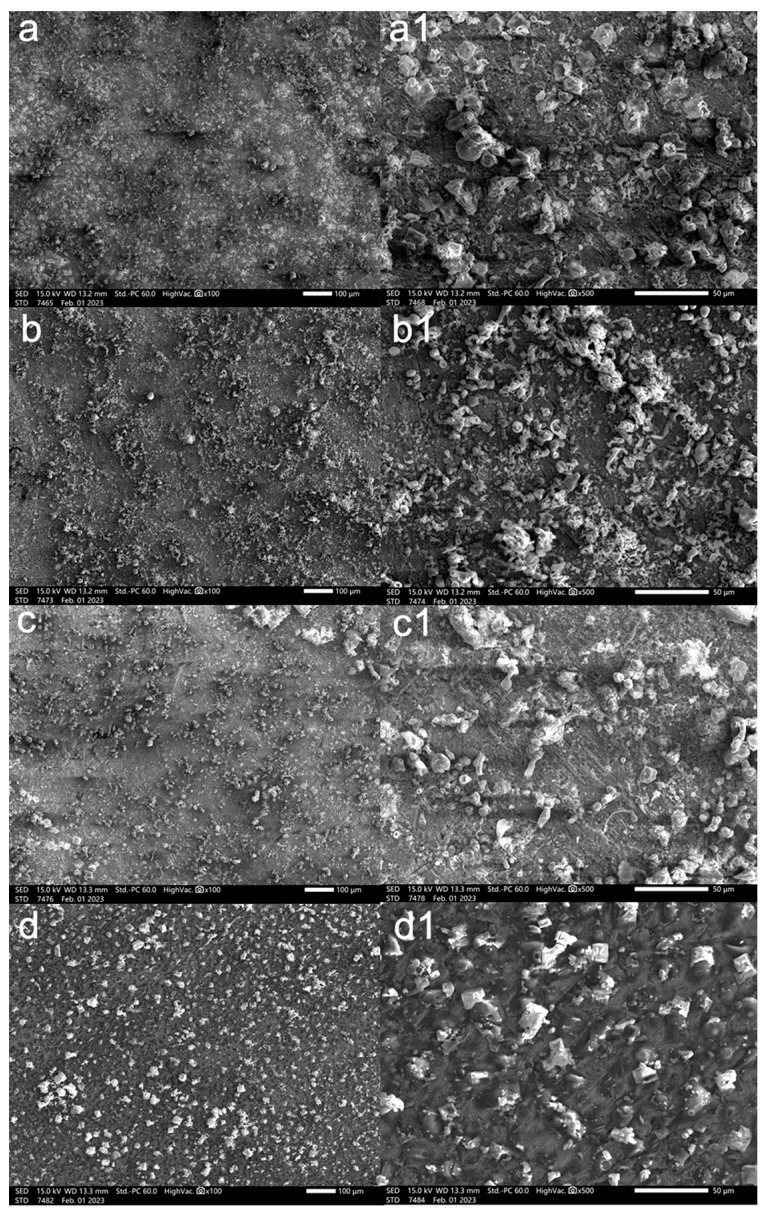
SEM images at 100 μm scale of L360N carbon steel in the CO_2_-saturated 5% NaCl corrosion medium without or with 100 ppm inhibitors at 40 °C for 72 h: (**a**) blank, (**b**) FB, (**c**) MG, (**d**) CV. (**a1**–**d1**) are at 50 μm scale, respectively.

The surface morphology of samples exposed to the corrosion medium at 40 °C with or without 100 ppm inhibitors is presented in Figure 6. It is evident that significant non-uniform corrosion is observed on the uninhibited sample, with a large amount of corrosion products (Figure 6a). For the system added with the corrosion inhibitor, a certain degree of corrosion can be observed. It is evident that CV performs better than MG, which in turn is superior to FB in terms of corrosion inhibition. Numerous crystals are present on the surface of CV-treated specimens due to the direct drying treatment of the sample surface after removal, which are attributed to sodium chloride crystals. In the presence of CV, the fine scratches produced during the grinding process can still be observed on the specimen surface, possibly indicating that CV exhibits the best corrosion inhibition performance [39].

#### 3.4.2. XPS Analysis

**Figure 7:**
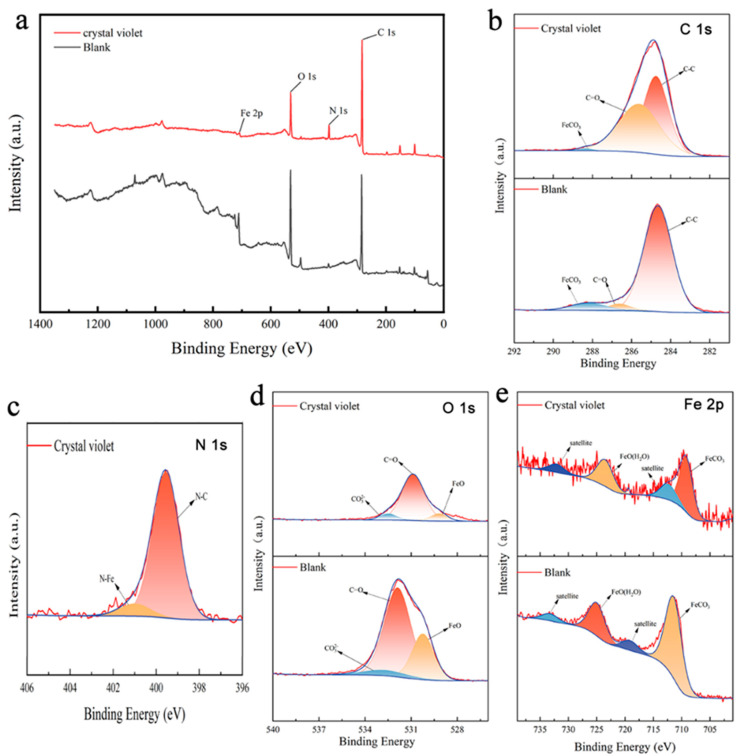
XPS high-resolution spectra of L360N carbon steel in the CO_2_-saturated 5% NaCl corrosion medium without or with 100 ppm inhibitors at 40 °C for 72 h: (**a**) full XPS spectra, (**b**) C 1s, (**c**) N 1s, (**d**) O 1s MG, (**e**) Fe 2p.

Figure 7 presents the XPS spectra of the sample after immersion in the CO_2_-saturated 5% NaCl corrosion without or with 100 ppm CV at 40 °C for 72 h. The full spectrum of XPS (Figure 7a) indicates the presence of C, O, N, and Fe elements. The elements of C, O, and Fe are present as expected; however, an increased amount of N is attributed to CV adsorbed on the surface of the sample. The high-resolution XPS spectra for C 1s, N 1s, O 1s, and Fe 2p, as well as the bonding configurations for each peak, are displayed in Figure 7b–e. The C 1s spectrum (Figure 7b) can be deconvoluted into peaks at 284.7, 286.6, and 288.2 eV, corresponding to C-C, C=O, and FeCO_3_, respectively [40]. For the N 1s spectrum (Figure 7c), the peak at 399.1 eV is attributed to the C-N in the corrosion inhibitor molecule, while the peak at 399.9 eV corresponds to N-Fe [41,42]. The O 1s spectrum features three peaks at 530.2, 531.9, and 532.7 eV, which are assigned to FeO, C=O, and CO_3_^2−^, respectively [43,44]. In the Fe 2p spectrum, two typical Fe2p1/2 and Fe 2p3/2 peaks are observed at binding energies of 725.0 eV and 709.3 eV, respectively. According to previous studies referenced in the literature [40,45,46], the peak located at 709.3 eV of Fe 2p3/2 is assigned to FeCO_3_. Additionally, satellite features associated with iron are present at binding energies of 719.1 eV and 733.2 eV. Furthermore, the Fe2p1/2 spectral feature at 725.0 eV is associated with the presence of FeO(H_2_O) [47]. Therefore, the XPS analysis confirms that CV has been successfully adsorbed onto the carbon steel surface.

#### 3.4.3. CLSM Analysis

**Figure 8:**
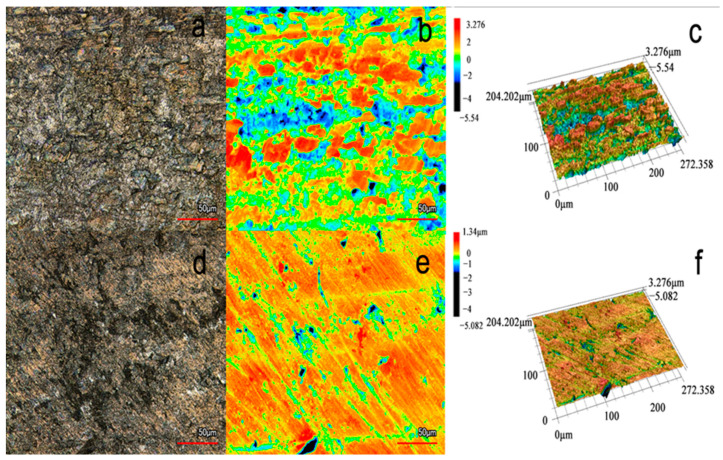
Three-dimensional image and height distribution map of L360N carbon steel obtained by CLSM test in the CO_2_–saturated 5% NaCl corrosion medium at 40 °C for 72 h: (**a**–**c**) without inhibitor; (**d**–**f**) with 100 ppm CV.

Figure 8 presents three-dimensional images of the L360N carbon steel with and without CV after being exposed for 72 in the CO_2_-saturated 5% NaCl solution environment. It is observed from Figure 8a–c that L360N surfaces are significantly compromised, with 3D imagery highlighting “mountain peak”-shaped corrosive deposits adorning the metal surface without any corrosion inhibitor. In contrast, Figure 8d–f display a markedly smoother and more homogenous surface, with only minor depressions and pits in morphology. This improvement is attributed to the inclusion of 100 mg/L CV, which appears to greatly slow the corrosion process.

Table 5 catalogs various parameters such as the arithmetic mean height (S_a_), maximum height (S_z_), texture aspect ratio (S_tr_), arithmetic mean peak curvature (S_pc_), and developed interfacial area ratio (S_dr_). From the surface roughness, we can more intuitively see that the roughness without the inhibitor (0.929 µm) is significantly higher than that with the inhibitor (0.277 µm) [48,49]. At the same time, for the sample with CV added, its other three-dimensional profile parameters are also much lower than those of the steel sample without the corrosion inhibitor except for S_tr_. The S_tr_ parameter represents the ratio of the size of the surface texture in the principal texture direction to the size in the direction perpendicular to it. A higher S_tr_ value indicates that the surface texture elements (such as scratches and grooves) are more pronounced in the principal texture direction. This indicates that the corrosion of carbon steel in the CO_2_–saturated 5% NaCl solution is very severe and that the addition of CV can significantly mitigate the impact of this corrosion.

### 3.5. Quantum Chemical Calculations

**Figure 9:**
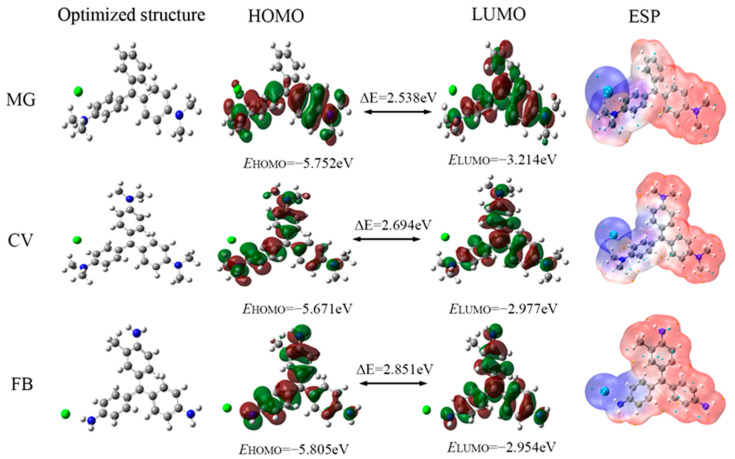
The obtained optimized structure, HOMO, LUMO, and electrostatic potential map (ESP) of three dyes by DFT.

Figure 9 presents optimized molecular structures of the inhibitors and their LUMO and HOMO distribution. HOMO and LUMO can determine the capacity of the molecule to accept electrons and provide electrons, respectively. The HOMO and LUMO distribution of three dyes are similar and around the benzene ring and N atoms, indicating that these structures can provide electrons to Fe atoms and can accept electrons from the Fe atoms. In addition, the values of EHOMO, E LUMO, and ΔE for MG, MV, and FB are shown in Figure 9. Typically, a higher EHOMO value indicates more reactive electrons within the bonding orbitals and an enhanced capability of the corrosion inhibitor molecules to donate electron pairs. Conversely, a smaller ELUMO value signifies a reduction in the system’s energy following the transfer of electron pairs to the bonding orbitals, facilitating the acceptance of electrons by the corrosion inhibitor [50]. The highest EHOMO (−5.671 eV) of CV surpasses those of MG and FB, which are −5.752 eV and −5.805 eV, respectively, signifying a superior electron-donating characteristic for CV. Furthermore, the ELUMO of MG at −3.214 eV is lower than that of CV at −2.977 eV and FB at −2.945 eV, indicating that MG possesses a more pronounced electron-accepting capability. It is clear that all dye molecules have positive ΔE values, which suggests that triphenylmethane dyes could donate π–electrons or lone pair electrons to the iron atoms. The smallest energy gap ΔE of MG indicates that it forms chemical bonds more easily in the adsorption process [40].

The van der Waals (vdW) ESP distribution is usually used to predict electrophilic and nucleophilic reaction sites [22], and the ESP distribution of three dyes are present in Figure 9. The red and blue surfaces illustrate the positively and negatively charged regions, respectively. The orange and cyan spheres indicate the maxima and minima of the ESP points, respectively. These structures, near the extreme points of ESP, are the most likely sites for chemical reaction. As can be seen in Figure 9, many extreme points are distributed around the benzene ring and N atoms, which indicates that the two regions are more inclined to be sites of electrophilic or nucleophilic reactions for dye molecules with Fe atoms [1].

### 3.6. MD Simulation

**Figure 10:**
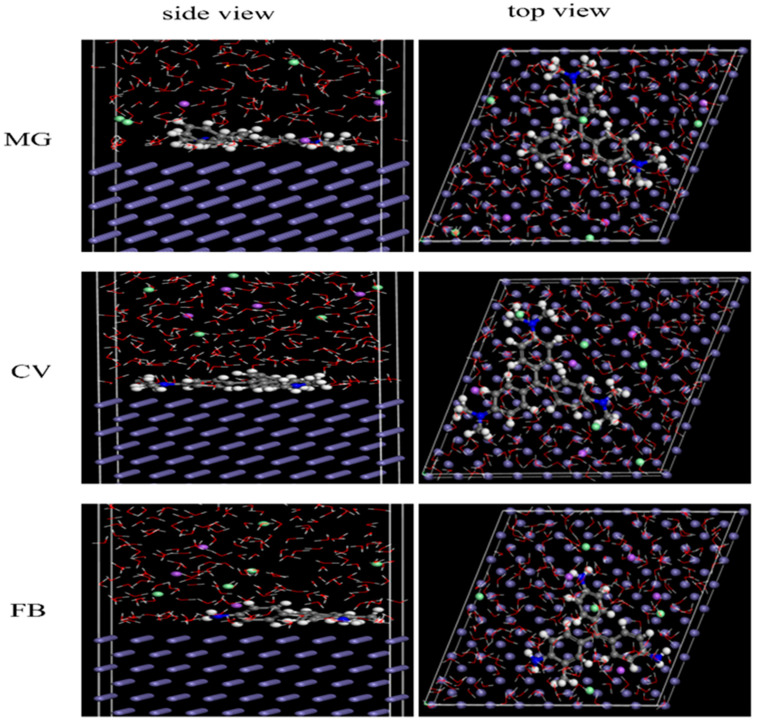
Side and top views of equilibrium adsorption configuration of the three adsorbed dye molecules onto the Fe (110) surface.

The interaction between the dye molecules and Fe surface is demonstrated by the MD simulation to further clarify the adsorption mechanism. Figure 10 shows the side and top views of dye adsorption configurations on the Fe (110)/H_2_O interface system. From Figure 10, dyes are adsorbed onto the metal surface in place of water, which creates a barrier to prevent corrosion. All dyes are absorbed almost parallel to the surface of Fe from the top view. According to Mulliken charges, electrons of the dye molecules’ N atom and aromatic ring are shared with Fe to create dative bonds through chemical adsorption [51]. Compared to Figure 10, the CV molecule is most parallel to the iron surface, which makes it highly effective at preventing corrosion. Table 6 displays the data for the adsorption locator’s outputs and descriptor. The obtained Eads values are −187.447, −215.611, and −163.795 kcal/mol for MG, CV, and FB, respectively. The negative value for Eads indicates that the inhibitors’ adsorption on the surface of Fe is spontaneous adsorption [40]. The largest negative adsorption energy value of CV compared to other dyes shows that it exhibits a strong interaction by the Fe surface, which further explains its dominant role in preventing corrosion.

### 3.7. Inhibition Mechanism of Triphenylmethane Dyes

**Figure 11:**
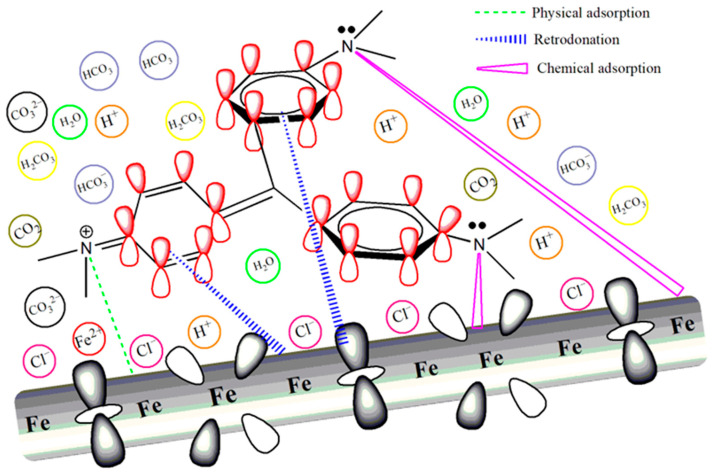
Schematic representation for corrosion inhibition mechanism of CV on L360N carbon steel.

Despite the abundance of research focused on the corrosion inhibition properties of dyes, there has been relatively scant attention paid to a detailed examination of their underlying inhibition mechanisms [12]. Based on the experimental and theoretical calculation results, a possible adsorption mechanism for triphenylmethane dyes is proposed. The corrosive solution is saturated with carbon dioxide containing 5% NaCl, and it is rich in species such as H_2_O, HCO_3_^−^, CO_2_, H_2_CO_3_, and Cl^−^ (Figure 11), which can severely corrode the surface of carbon steel. The anodic process involves the dissolution of Fe, while the cathodic process involves the reduction of H^+^ and H_2_CO_3_ [1,43]. Upon the addition of the dye, surface analysis through XPS and theoretical calculations suggests that the dye can adsorb onto the steel surface both physically and chemically. The inhibition mechanism encompasses a dual strategy involving both physical adsorption (through electrostatic attraction) and chemical adsorption (involving the sharing of electron density), as depicted in Figure 11. In the electrolyte solution, this orientation of negatively charged Cl^−^ toward the solution interface promotes the physical adsorption of N^+^ in the dye molecule [51]. As for chemisorption, it is proposed that the inhibitors adhere to the surface through the interaction between the lone pairs of electrons on the N atom or the π-electrons from the aromatic benzene ring in the dye molecule with the unoccupied d-orbitals of iron atoms [28]. CV exhibits the best adsorption effect, attributed to its multiple electron-donating groups, which enhance the electron-donating capability of its benzene rings.

## 4. Conclusions

In conclusion, our investigation demonstrates that among the triphenylmethane dyes studied as corrosion inhibitors for L360N carbon steel in the CO_2_-saturated 5% NaCl corrosion medium, CV exhibits the most effective corrosion inhibition for carbon steel, outperforming MG and FB. Electrochemical assessments clearly categorize CV as a mixed-type corrosion inhibitor, which conforms to the Langmuir adsorption isotherm, suggesting a combination of physical and chemical adsorption mechanisms. Surface analysis techniques including SEM, XPS, and CLMS have validated the substantial improvement in the corrosion resistance of carbon steel in the presence of CV. Furthermore, quantum chemical calculations and molecular dynamics simulations have provided insight at the molecular level to understand the inhibitors on the surface of carbon steel, affirming the primary adsorption sites, adsorption behavior, and adsorption energies of dyes. This research offers a strong foundation for the application of triphenylmethane dyes as effective corrosion inhibitors in relevant industrial settings.

## Figures and Tables

**Table 1:** Weight loss measurements for L360N carbon steel exposed to CO_2_-saturated NaCl corrosion medium at 40 °C.

Inhibitors	Concentration (ppm)	Corrosion Rate (mm/y)	Inhibition Efficiency (%)
Blank	0	0.323 ± 0.005	—
CV	100	0.050 ± 0.002	82.8 ± 0.3
MG	100	0.102 ± 0.004	64.1 ± 0.7
FB	100	0.143 ± 0.002	49.8 ± 0.5

**Table 2:** Fitted electrochemical parameters deduced from polarization curves for L360N carbon steel in the CO_2_-saturated 5% NaCl corrosion medium with different concentrations of CV at 40 °C.

Temperature (°C)	Concentration (ppm)	*i*_corr_ (A/cm^2^)	*E*_corr_ (V vs. SCE)	*Ƞ*_p_ (%)
25	0	(5.62 ± 0.15) × 10^−5^	−0.714 ± 0.003	
	25	(2.36 ± 0.08) × 10^−5^	−0.709 ± 0.002	58.01 ± 0.03
	50	(7.33 ± 0.21) × 10^−6^	−0.708 ± 0.002	86.96 ± 0.01
	75	(4.06 ± 0.03) × 10^−6^	−0.735 ± 0.001	92.78 ± 0.01
	100	(2.90 ± 0.21) × 10^−6^	−0.774 ± 0.002	94.84 ± 0.01
	150	(2.88 ± 0.18) × 10^−6^	−0.758 ± 0.002	94.89 ± 0.01
40	0	(1.33 ± 0.31) × 10^−4^	−0.712 ± 0.003	
	25	(5.82 ± 0.11) × 10^−5^	−0.713 ± 0.001	56.13 ± 0.03
	50	(7.45 ± 0.13) × 10^−5^	−0.706 ± 0.004	71.31 ± 0.02
	75	(3.51 ± 0.25) × 10^−5^	−0.780 ± 0.002	73.54 ± 0.01
	100	(1.48 ± 0.35) × 10^−5^	−0.694 ± 0.002	88.87 ± 0.01
	150	(7.15 ± 0.26) × 10^−6^	−0.756 ± 0.001	94.61 ± 0.01
60	0	(2.71 ± 0.24) × 10^−4^	−0.711 ± 0.003	
	25	(1.67 ± 0.05) × 10^−4^	−0.714 ± 0.002	38.29 ± 0.03
	50	(7.94 ± 0.18) × 10^−5^	−0.714 ± 0.002	70.72 ± 0.02
	75	(7.88 ± 0.06) × 10^−5^	−0.715 ± 0.001	70.93 ± 0.02
	100	(6.64 ± 0.19) × 10^−5^	−0.707 ± 0.002	75.51 ± 0.01
	150	(1.91 ± 0.14) × 10^−5^	−0.703 ± 0.003	92.94 ± 0.01

**Table 3:** The inhibition efficiencies of studied dyes and other dyes reported in the literature.

Dye Molecules	Medium	Material	Concentration	Temperature	η %	Method	Ref.
Crystal violet	CO_2_-saturated 5% NaCl solution	L360N	150	25	94.9	PDP	This work
Crystal violet	CO_2_-saturated 5% NaCl solution	L360N	100	40	82.8	WL	This work
Malachite green	CO_2_-saturated 5% NaCl solution	L360N	100	40	64.1	WL	This work
Fuchsine basic	CO_2_-saturated 5% NaCl solution	L360N	100	40	49.8	WL	This work
Malachite green	1.0 mol/L HCl	AA1060	360	30	77.8	WL	[16]
Crystal violet	0.5 mol/L H_2_SO_4_	mild steel	4	30	56.7	WL	[32]
Methyl green	0.5 mol/L H_2_SO_4_	low carbon steel	400	60	73	WL	[33]
Fuchsine basic	1.0 mol/L HCl	steel	200	25	99.0	EIS	[34]
Colocid dye	1.0 mol/L HCl	mild steel	600	25	89.3	WL	[35]

**Table 4:** Thermodynamic parameters of CV exposed to CO_2_-saturated NaCl corrosion medium at 40 °C (313.15 K).

Inhibitors	Slope	Intercept (mg/L)	K_abs_ (L/mg)	$∆G_{\mathrm{abs}}^{0}$ (KJ/mol)	Linear Correlation Coefficient, R^2^
CV	0.896	25.887	0.0386	−27.496	0.99

**Table 5:** Surface roughness parameters of CLSM images for L360N carbon steel without and with 100 ppm CV in the CO_2_-saturated 5% NaCl corrosion medium at 40 °C for 72 h.

Surface	S_a_ (µm)	S_z_ (µm)	S_tr_	S_pc_ (mm^−1^)	S_dr_
without CV	0.929	8.815	0.319	7039.788	0.7405
with CV	0.277	6.476	0.642	5710.048	0.3936

**Table 6:** Outputs and descriptors calculated from MD simulations for dyes on Fe (110)/H_2_O system.

	*E*_T_ (kcal/mol)	*E*_Surf+Sol_ (kcal/mol)	*E*_Inh+Sol_ (kcal/mol)	*E*_Sol_ (kcal/mol)	*E*_ads_ (kcal/mol)
MB	−3159.43	−2860.45	−2694.54	−2583.02	−187.447
CV	−3198.34	−2899.12	−2734.55	−2650.94	−215.611
FB	−3235.43	−2895.63	−2807.53	−2631.53	−163.795

## Data Availability

The original contributions presented in the study are included in the article, further inquiries can be directed to the corresponding author.
